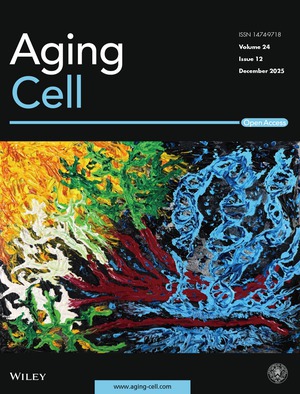# Additional Cover

**DOI:** 10.1111/acel.70322

**Published:** 2025-12-08

**Authors:** Rikuou Yokosawa, Kentaro Noma

## Abstract

Cover legend: The cover image is based on the article *A Nuclear Hormone Receptor nhr‐76 Induces Age‐Dependent Chemotaxis Decline in C. elegans* by Rikuou Yokosawa et al., https://doi.org/10.1111/acel.70277.